# Gallbladder carcinoma: an initial clinical experience of reduced field-of-view diffusion-weighted MRI

**DOI:** 10.1186/s40644-020-00326-x

**Published:** 2020-07-17

**Authors:** Sisi Wu, Xianlun Zou, Qiuxia Wang, Daoyu Hu, Zhen Li, Chuou Xu

**Affiliations:** Department of Radiology, Tongji Hospital, Tongji Medical College, Huazhong University of Science and Technology, 1095 Jiefang Avenue, Qiaokou district, Wuhan, 430030 Hubei China

**Keywords:** Diffusion-weighted imaging, Full field-of-view, Gallbladder carcinoma, Image quality, Reduced field-of-view

## Abstract

**Background:**

The purpose of this study is to compare the diagnostic value, imaging quality and apparent diffusion coefficient (ADC) value of reduced field-of-view diffusion-weight imaging (r-FOV DWI) and full field-of-view diffusion-weight imaging (f-FOV DWI) in patients with gallbladder carcinoma and other lesions of gallbladder.

**Methods:**

Two hundred ninety-six patients with gallbladder diseases underwent both r-FOV DWI and f-FOV DWI on a 3.0 T MRI scanner. Two radiologists assessed subjective image quality parameters independently. The Wilcoxon signed-rank test was used to compare subjective qualitative image score. Objective quality values and the mean ADC values were analyzed by paired *t*-test. The correlation between pathological results and mean ADC value were estimated using Spearman rank correlation analysis.

**Results:**

The CNR value (10.23 ± 2.92) and image quality score (13.84 ± 1.07) of r-FOV DWI were significantly higher than those of f-FOV DWI (5.24 ± 1.29 ***P***<0.001; 10.41 ± 1.11 P<0.001). There was no significant difference between mean ADC values of the two DWI sequences for all three groups (Group 1, chronic cholecystitis; Group 2, benign lesions of gallbladder; Group 3, gallbladder carcinoma. *P* = 0.239, 0.974 and 0.226 respectively). For both DWI sequences, the mean ADC values were the highest in the group of cholecystitis and the lowest in the group of gallbladder carcinoma (2.49 ± 0.14 vs 1.49 ± 0.12; 2.50 ± 0.14 vs 1.50 ± 0.13, for f-FOV and r-FOV respectively), the differences among groups were statistically significant (*P*<0.01). The mean ADC values for both DWI sequences were negatively correlated with the group number, which increased with the malignant tendency of lesions (*r* = − 0.892, P<0.01; r = − 0.913, P<0.01 for f-FOV and r-FOV respectively).

**Conclusion:**

Reduced Field-of-view Diffusion-weighted MRI is a good tool to diagnosis the gallbladder carcinoma, with better image quality and without affecting ADC values.

## Background

Primary gallbladder carcinoma is a common malignant tumor of the biliary system with high lethal malignancy and poor prognosis. Most cases of gallbladder carcinoma are diagnosed postoperatively, which are called occult gallbladder carcinoma. Early detection and treatment of gallbladder cancer can improve the prognosis. Furthermore some benign lesions, such as gallbladder adenoma and gallbladder polyps, have risk of developing malignancy and also require early detection and treatment [1]. However, a reported 5-year survival rate of advanced gallbladder cancer is merely 3% [2]. Therefore, early detection and differentiation benign lesions from malignant lesions for gallbladder are of great significance for clinical treatment.

Over the past few decades, ultrasonography was considered as one of the most important imaging modality for detection of gallbladder diseases. However, it is limited in the diagnosis of early lesion because of the lower sensitivity and specificity [3]. Recently, diffusion weighted imaging (DWI) has been reported to facilitate the diagnosis of gallbladder cancer, and it can also differentiate gallbladder benign and malignant disorders [4–6]. The apparent diffusion coefficient (ADC) value of DWI can indicate the histological grade of primary gallbladder carcinoma [7].

Currently, the standard sequence used for clinical DWI is full filed-of-view (f-FOV) single-shot echo-planar imaging (SS-EPI). Due to its long readout time and low bandwidth in the phase-encode direction, SS-EPI is prone to artifacts, distortion and blurring, which affects the accuracy of the detection of small lesions [8]. The reduced field-of-view (r-FOV) DWI using two-dimensional spatially selective excitation and a 180° refocusing pulse to reduce the FOV in the phase-encode direction, not only provides fewer artifacts, but also higher quality images [9]. This technique has been widely used in spinal cord, rectum, urinary bladder and prostate [8–11]. However, to the best of our knowledge, no previous studies have been reported in the detection of gallbladder diseases using r-FOV DWI. The purpose of this study is to compare the diagnostic value, imaging quality and apparent diffusion coefficient (ADC) value of reduced field-of-view diffusion-weight imaging (r-FOV DWI) and full field-of-view diffusion-weight imaging (f-FOV DWI) in patients with gallbladder carcinoma and other lesions of gallbladder, and also to determine whether the ADC values of gallbladder lesions for both DWI sequences are correlated with the pathological results.

## Material and methods

### Patients

**Fig. 1:**
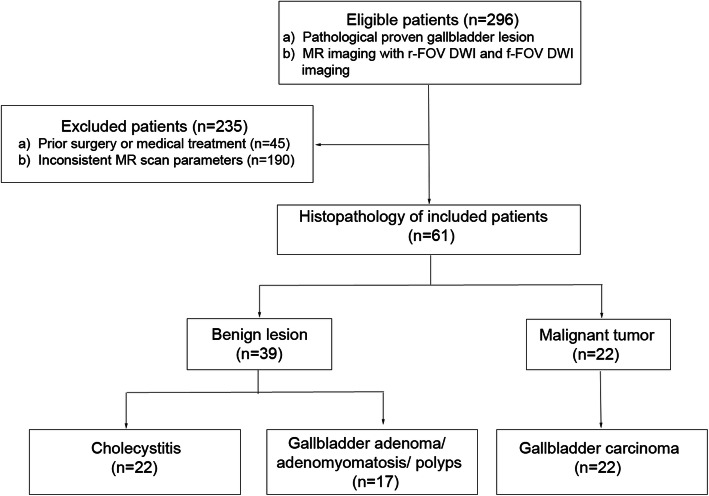
Flow diagram demonstrating patient and tumor characteristics

This retrospective study was approved by the Institutional Review Board of our institution and requirement for written informed consent was waived. Between November 2016 and January 2019, 296 patients were enrolled in this study, the inclusion criteria were employed as follows: 1) all patients having pathological results of gallbladder lesions; 2) patients underwent pretreatment MRI for both f-FOV and r-FOV DWI imaging. And the exclusion criteria were: 1) patients receiving previous surgery or medical treatment; 2) patients underwent MR scanning with inconsistent scan parameters. Among the 296 patients, 45 patients were excluded from this study because of previous surgery or medical treatment, 190 patients were excluded due to inconsistent MR scan parameters. As a result, 61 patients were included in this study (28 males; 33 females; mean age 55.80 ± 11.63 years; range 25–78 years). All the lesions were confirmed by pathological results, including 22 cases of chronic cholecystitis, 22 cases of gallbladder carcinoma, 7 cases of gallbladder adenomyomatosis, 7 cases of gallbladder adenoma and 3 cases of gallbladder polyps (Fig. 1).

### MRI data acquisition

All patients underwent magnetic resonance imaging (MRI) studies on a GE 3.0 T Discovery 750 scanner with a 32-channel torso phase-array coil. All patients fasted for at least 6 h and had breathing training before scanning. Breath gating was employed during MRI scanning.

The diffusion-weighted SS-EPI f-FOV and r-FOV images were acquired in the axial plane, focusing on lesions. The scanning parameters of the SS-EPI DWI (f-FOV DWI) were as follows: TR = 3157 ms, TE = 57.5 ms, FOV = 36 mm × 28.8 mm, matrix = 160 × 128, slice thickness = 4 mm, gap = 1 mm, bandwidth = ±250KHz, NEX = 10, and b = 0 and 800 s/mm2. The scanning parameters of r-FOV DWI were as follows: TR = 3000 ms, TE = 55.3 ms, FOV = 20 mm × 10 mm, matrix = 128 × 64, slice thickness = 4 mm, gap = 1 mm, bandwidth = ±250KHz, NEX = 10, and b = 0 and 800 s/mm^2^ (Table 1).

**Table 1:** Imaging Parameters for f-FOV and r-FOV Diffusion-Weighted Imaging

Sequence parameter	f-FOV	r-FOV
TR/TE (msec)	3157/57.5	3000/55.3
Thickness/Gap (mm)	4/1	4/1
FOV (mm)	360 × 288	200 × 100
Matrix	160 × 128	128 × 64
NEX	10	10
Bandwidth (KHZ)	250	250
b-value (sec/mm^2^)	0, 800	0, 800

*TR* repetition time, *TE* echo time, *FOV* field of view, *f-FOV* full FOV, *r-FOV* reduced FOV, *NEX* number of excitations

### Image analysis

#### Objective analysis

All the images were reviewed and analyzed on picture archiving and communication system (PACS) and GE workstation 4.6. The r-FOV and f-FOV images were blindly assessed by two independent radiologists with 6 years and 10 years of imaging experience, signal intensity, background signal intensity, and intercostals signal intensity were measured and calculated. The signal-to-noise ratio (SNR) and cintrast-to-noise (CNR) were calculated as the following formulas:

$$ \mathrm{SNR}=\frac{{\mathrm{S}}_{\mathrm{lesion}}}{{\mathrm{S}\mathrm{D}}_{\mathrm{background}}}\kern0.5em \mathrm{CNR}=\frac{\underline {\left|{\mathrm{S}}_{\mathrm{lesion}}\hbox{-} \left.{\mathrm{S}}_{\mathrm{tissue}}\right|\right.}}{\sqrt{{{\mathrm{S}\mathrm{D}}_{\mathrm{lesion}}}^2+}{{\mathrm{S}\mathrm{D}}_{\mathrm{tissue}}}^2} $$

The SNR was calculated as the ratio between the mean signal intensity inside the lesion (S_lesion_) and the standard deviation of background noise (SD_background_); The CNR was defined as the ratio of the mean signal intensity difference between lesion (S_lesion_) and normal tissue (S_tissue_) divided by the standard deviation of the lesion (SD_lesion_) and normal tissue (SD_tissue_) .

All of the ADC values were calculated on the GE workstation 4.6 with a standard software package. The ADC value of a lesion was measured by carefully drawing a region of interest (ROI) around the largest area of the lesion for adjacent three sections (b = 800 s/mm^2^) with homogenous signal intensity, without artifacts or deformations. The lesion was identified on b = 0 s/mm^2^. Areas containing bile, blood, cystic degeneration or necrosis were avoided. This procedure was performed 3 times for each patient by a single observer. The average of 3 measurements was taken as the average ADC value.

#### Subjective analysis

The r-FOV DWI and f-FOV DWI images were evaluated by two experienced radiologists based on the following characteristics: sharpness, distortion, artifacts and lesion conspicuity, scoring was based on a 4-point scale as follows: sharpness (1 = poor, 2 = fair, 3 = good, 4 = excellent); distortion (1 = severe distortion, 2 = moderate distortion, 3 = slight distortion, 4 = no distortion); artifacts (1 = severe artifacts, may interfere the diagnostic information, 2 = severe artifacts, may partially interfere the diagnostic information, 3 = slight artifacts, no interfere the diagnostic information, 4 = no artifacts); lesion conspicuity (1 = poor, considered unrecognized, 2 = fair, most of the outlines unclear, 3 = good, small part of outline unclear, 4 = excellent, clear outline). Total subjective image quality score was calculated by adding the above four values all together in the same imaging section.

### Statistical analysis

All analyses were performed using SPSS for Windows, version 19.0 (IBM, Armonk, NY). The interobserver variability of objectively rated image quality (including CNR and SNR) was assessed using the intraclass correlation coefficient (ICC) test (0.00–0.20, poor agreement; 0.21–0.40, fair agreement; 0.41–0.60, moderate agreement; 0.61–0.80, good agreement; 0.81–1.00, excellent agreement). The interobserver variability of subjective image quality score (including sharpness, distortion, artifacts and lesion conspicuity) was evaluated by weighted kappa statistics (0.00–0.20, poor agreement; 0.21–0.40, fair agreement; 0.41–0.60, moderate agreement; 0.61–0.80, good agreement; 0.81–1.00, excellent agreement). The Wilcoxon signed-rank test was used to compare the subjective qualitative image scores (sharpness, distortion, artifacts and lesion conspicuity) between r-FOV DWI and f-FOV DWI. Objective quality values (SNR, CNR) and the mean ADC values were statistically analyzed by paired *t*-test for the two DWI sequences, and the correlation between pathological results and mean ADC value was estimated using Spearman rank correlation analysis. Results with a *P* value less than 0.05 were considered statistically significant.

## Results

**Fig. 2:**
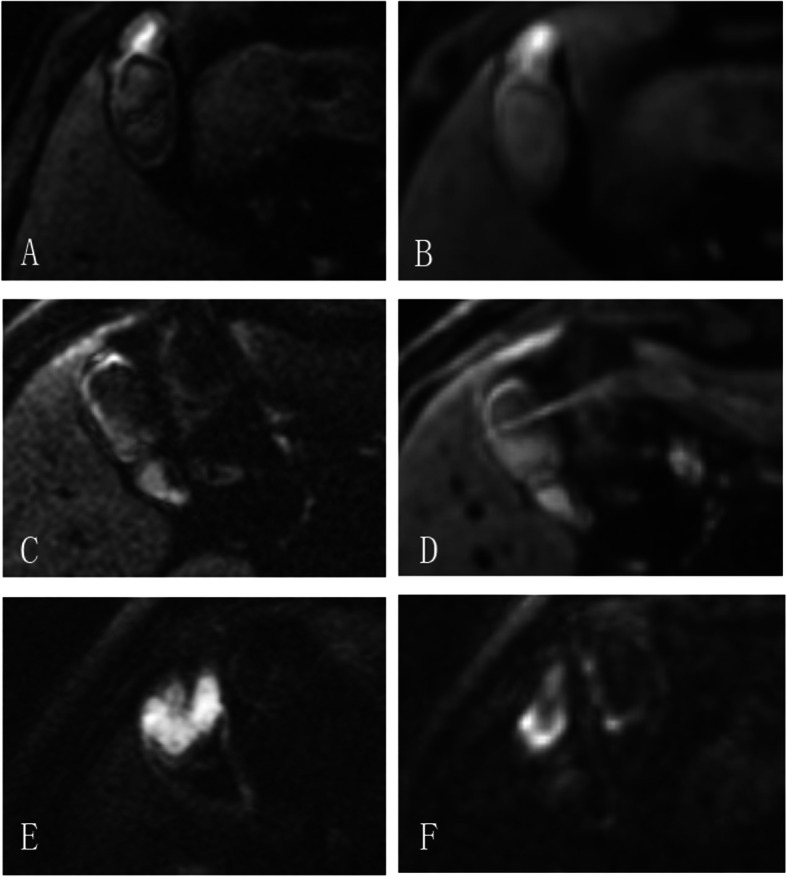
Images for subjective quality assessment. **a**, **b**: Assessment of sharpness and lesion conspicuity (gallbladder adenomyomatosis). **a**: r-FOV DWI image shows the soft tissue signal at the bottom of the gallbladder with a sharp margin, and the lesion features are clearly demonstrated. Subjective image quality scores of sharpness and lesion conspicuity are both 3. **b**: f-FOV DWI image shows the soft tissue signal at the bottom of the gallbladder with blurred edges and lower contrast comparing with Fig. A. The subjective image quality scores of sharpness and lesion conspicuity are both 2. **c**, **d** (gallbladder adenoma): Assessment of artifacts. **c**: r-FOV DWI image shows mild artifacts around the wall of the gallbladder. Subjective image quality score of artifacts is 4. **d**, f-FOV DWI image shows obvious artifacts. Subjective image quality score of artifacts is 3. **e**, **f** (gallbladder adenocarcinoma): Assessment of distortion. **e**: r-FOV DWI image shows a diffusion-restricted lesion at the bottom of the gallbladder without distortion. Subjective image quality score of distortion is 4. **f**: f-FOV DWI image shows the edge of the lesion with obvious distortion. Subjective image quality score of distortion is 2

1. Pathological Results
  Of all 61 patients, the pathological results revealed 22 cases (36.06%) of gallbladder carcinoma, 7 cases (11.48%) of gallbladder adenoma, 7 cases (11.48%) of gallbladder adenomyomatosis, 3 cases (4.92%) of gallbladder polyps and 22 cases (36.06%) of cholecystitis. These cases were divided into 3 groups according to the malignant tendency of the lesion (Group 1, chronic cholecystitis; Group 2, benign lesions of gallbladder including gallbladder adenomyomatosis, gallbladder adenoma and gallbladder polyps; Group 3, gallbladder carcinoma), the larger the group number was, the more likely the lesion tends to be malignant.
2. Interobserver Variability of Image Quality
  Objective image quality values (SNR, CNR) of both DWI methods had good to excellent agreement. The ICC values ranged from 0.776 to 0.967. All of the subjective image quality scores (sharpness, distortion, artifacts and lesion conspicuity) had good to excellent agreement, the κ values between two radiologists ranged from 0.769 to 0.873 (Table 2).
3. Comparison of Image Quality
  Based on the formula of image resolution = FOV/matrix, the image resolution of f-FOV DWI and r-FOV DWI was 2.25 × 2.25 mm and 1.56 × 1.56 mm respectively. The comparison of image quality scores assessed by two radiologists between f-FOV and r-FOV DWI is shown in Table 3. The CNR value of r-FOV DWI was significantly higher than that of f-FOV DWI (10.23 ± 2.92, 5.24 ± 1.29, ***P***<0.001) while the SNR value of r-FOV DWI was lower than that of f-FOV DWI. The subjective image quality scores of sharpness (3.43 ± 0.47, 2.64 ± 0.59, ***P***<0.001), distortion (3.35 ± 0.61, 2.59 ± 0.54, ***P***<0.001), artifacts (3.66 ± 0.46, 2.75 ± 0.54, ***P***<0.001), lesion conspicuity (3.39 ± 0.55, 2.48 ± 0.62, ***P***<0.001) and total subjective image quality score (13.84 ± 1.07, 10.41 ± 1.11, ***P***<0.001) for r-FOV DWI were significantly higher than those for f-FOV DWI (Fig. 2).
4. Quantitative Assessment of ADC values of Gallbladder lesions

**Table 2:** Interobserver Variability of Image Quality of f-FOV and r-FOV Diffusion-Weighted Imaging

Image parameter	f-FOV	r-FOV
SNR	0.967 (0.946—0.980)	0.938 (0.899—0.962)
CNR	0.776 (0.652—0.859)	0.878 (0.804—0.925)
Sharpness	0.769 (0.606—0.908)	0.833 (0.689—0.965)
Distortion	0.873 (0.751—0.969)	0.799 (0.652—0.920)
Artifacts	0.837 (0.686—0.966)	0.855 (0.691—0.967)
Lesion conspicuity	0.826 (0.682—0.942)	0.846 (0.711—0.968)

Data in parentheses are 95% confidence intervals, SNR = signal-to-noise ratio, CNR = contrast-to-noise ratio

**Table 3:** Comparison of Image Qualities Between f-FOV and r-FOV Diffusion-Weighted Imaging

Image parameter	f-FOV	r-FOV	***P*** value
SNR	40.75 ± 10.30	22.34 ± 5.90	<0.001
CNR	5.24 ± 1.29	10.23 ± 2.92	<0.001
Sharpness	2.64 ± 0.59	3.43 ± 0.47	<0.001
Distortion	2.59 ± 0.54	3.35 ± 0.61	<0.001
Artifacts	2.75 ± 0.54	3.66 ± 0.46	<0.001
Lesion conspicuity	2.48 ± 0.62	3.39 ± 0.55	<0.001
Total subjective IQ	10.41 ± 1.11	13.84 ± 1.07	<0.001

Data are means±standard deviations (averages between two readers)

**Fig. 3:**
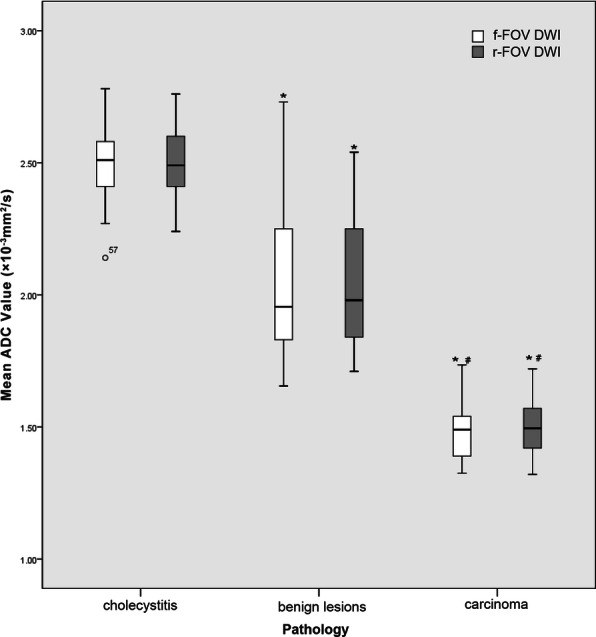
Mean ADC values of both f-FOV and r-FOV diffusion-weighted imaging related to pathological results. * *P*<0.01 versus the group of cholecystitis, ^#^*P*<0.01 versus the group of benign disease

For both DWI sequences, the mean ADC values in the group of cholecystitis were the highest (2.49 ± 0.14, 2.50 ± 0.14, respectively) while the mean ADC values in the group of malignant tumor were the lowest (1.49 ± 0.12, 1.50 ± 0.13, respectively), the differences among groups were statistically significant (*P*<0.01) (Fig. 3). There was no significant difference between mean ADC values of f-FOV DWI and r-FOV DWI (*P* = 0.625). There was no significant difference between mean ADC values of the two DWI sequences for cholecystitis, benign lesions or malignant tumor either (*P* = 0.239, 0.974 and 0.226 respectively). The ADC values of different pathological results of gallbladder lesions in the two DWI sequences are shown in Table 4. The mean ADC values for both DWI sequences were negatively correlated with the group number, which increased with the malignant tendency of lesions (*r* = − 0.892, P<0.01; r = − 0.913, P<0.01 respectively) (Table 5).

**Table 4:** Comparison of Apparent Diffusion Coefficient Values Between f-FOV and r-FOV Diffusion-Weighted Imaging Related to Pathological results

Pathology	ADC value (× 10^− 3^ mm^2^/s)		P value
	f-FOV	r-FOV	
1	2.49 ± 0.14	2.50 ± 0.14	0.239
2	2.05 ± 0.29	2.05 ± 0.24	0.974
3	1.49 ± 0.12	1.50 ± 0.13	0.226
Total	2.02 ± 0.46	2.02 ± 0.45	0.625

Data are means±standard deviations

**Table 5:** Spearman Rank Correlation Analysis of Apparent Diffusion Coefficient Values and Pathological Results

	*r* value	*P* value
f-FOV	−0.892(−0.933, -0.819)	<0.001
r-FOC	− 0.913(− 0.936, -0.857)	<0.001

Data in parentheses are 95% confidence intervals

## Discussion

Diffusion-Weighted imaging (DWI) is a non-invasive imaging technique which reflects changes in tissue cell density and microstructure [12]. Single-shot echo-planar imaging (SSEPI) technique was employed in DWI and it has a long readout time and a narrow bandwidth in phase encoding direction. SSEPI may cause image distortion, image blur and signal loss, it also contains bone, soft tissue, liquid, gas and other substances which make the field uneven, and artifacts will be produced easily because of the large range of full field-of-view (f-FOV) [11, 13, 14]. The r-FOV DWI technique in this study uses a 2D radiofrequency pulse to selectively excite the small volume of interest, such as gallbladder, thereby reducing number of baseline required for K-space filling, thus reducing readout time, so that the deformation and artifacts of images can be reduced effectively [14, 15].

In this study, the r-FOV DWI sequence provided a significantly better image quality. CNR and subjective image quality scores of r-FOV DWI sequence were significantly higher than those of f-FOV DWI, which are consistent with previous studies. Sapkota et al. ^9^demonstrated that r-FOV DWI image presented with higher resolution and significantly reduced distortion. Similar results were also noted in abdominal organs. Peng et al. [8] and Wang et al. [10] reported that image quality was significantly higher for r-FOV DWI images than the f-FOV DWI images in both rectal and urinary bladder tumors. Similar results were obtained in prostate tumor by Tamada et al [11] The r-FOW DWI sequence allowed the edge of the gallbladder wall and the structure of the tissue to be displayed more clearly, and it is easier to detect small lesions. However, the result of SNR was the opposite. Theoretically, SNR decreases as FOV reduces and image resolution increases, therefore, suitable reduced FOV, not smaller FOV is better at assessing gallbladder lesions [16].

In our study, there was no significant difference in the mean ADC values between two DWI sequences. Attenberger et al. [13] have shown that the ADC values of the prostate in different DWI sequences were not significantly different. Dong also had the same conclusion in breast [16]. Our results are consistent with above results. However, the conclusion obtained in thyroid gland, lesion and muscle in the head and neck was totally different [17, 18]. The reason for the different ADC values may be the lower resolution and poorer image quality of f-FOV DWI image, which affected by partial volume effect and magnetic sensitivity artifact when measuring the ADC value in the region of interest. The r-FOV DWI images have higher resolution, better fat saturation and fewer artifacts, thus the ADC values measured by r-FOV DWI is more closer to the genuine ADC value of the tissue.

In addition, the ADC value is affected by other factors, such as blood flow perfusion, b values of DWI and so on. The blood flow-rich organ is affected by blood flow, resulting error in the measurement of ADC values [19]. In our clinical protocol for abdominal DWI, the b-value was optimized to be 800 s/mm^2^ based on our experience, so we chose a b-value of 800 s/mm^2^ in r-FOV DWI in order to compare these results with those in f-FOV DWI. The blood perfusion of the gallbladder was not abundant, thus, the influence of blood perfusion was avoided. Also, we selected the region of tissue with uniform signal instead of the part with artifacts and deformation while drawing the ROI, and the area of ROI remained the same in the two DWI sequences, which may be the possible reason that the difference between the measured ADC values in the two DWI sequences was not statistical significant.

Furthermore, our study indicated r-FOV DWI can detect the gallbladder tumor among gallbladder lesions. The mean ADC values of malignant tumor of gallbladder (group 3) was significantly lower than that of benign lesions of gallbladder (group 2) and cholecystitis (group 1), the mean ADC values of benign lesions of gallbladder (group 2) was significantly lower than that of cholecystitis (group 1). The pathological results include gallbladder adenoma and gallbladder polyps in group 2, they are both belonged to gallbladder polypoid-lesions (GPs), which have been considered as premalignant lesions of gallbladder, studies showed that 15.3% of GPs lesser than10mm in size were malignant, while the lesion was greater than 10 mm, the malignant rate was increased [1, 20]. Therefore, the malignant trend of these three groups in increasing as the group number increases (in other words, the malignancy of group 1 was the lowest while group 3 was the highest). A significant tendency of a negative correlation between the mean ADC values and the pathological results was observed in both DWI sequences.

Our results are consistent with several prior studies. Kitazume et al. [5] demonstrated that the ADC value of malignant lesions was significantly lower than that of benign ones. Furthermore, Yoshioka et al. [21] showed that the ADC value was useful for differential diagnosis of inflammatory diseases, adenoma and cancer of the gallbladder. DWI reflects the random diffusion motion of water molecules, which are affected by cell density, microcirculation and histological composition, apparent diffusion coefficient (ADC) derived from DWI can be treated as a quantitative parameter [12]. Cancer cells in gallbladder proliferated rapidly, which leaded to the increasing of cell density, besides, the organelles, intracellular matrix and soluble macromolecules restricted the diffusion of water molecules, then the ADC values declined [21].

There are several limitations in this study. Firstly, the number of cases enrolled was relatively small, which could have resulted in selective bias of measured values. Secondly, the ROI drawing was based on the largest area of the lesion for adjacent three sections, which may not reflect the whole information of lesions, the whole volume measurement should be considered for further studies. Furthermore, benign gallbladder lesions (group 2) were not subdivided according to the specific pathological results. Further cohort studies should be performed with larger sample size and subdivision of pathological results to ensure the veracity and reliability.

## Conclusion

The image quality of r-FOV DWI was significantly higher than that of f-FOV DWI. There was no significant difference in the mean ADC values between these two DWI sequences, and r-FOV DWI is a good tool to diagnose the gallbladder carcinoma.

## Data Availability

Not applicable.

## Abbreviations

ADC: Apparent diffusion coefficient
r-FOV DWI: Reduced field-of-view diffusion-weight imaging
f-FOV DWI: Full field-of-view diffusion-weight imaging
DWI: Diffusion weighted imaging
SS-EPI: Single-shot echo-planar imaging
MRI: Magnetic resonance imaging
PACS: Picture archiving and communication system
SNR: Signal-to-noise ratio
CNR: Cintrast-to-noise.
ROI: Region of interest
